# Supplementary material for: Small molecule inhibitors reveal an indispensable scaffolding role of RIPK2 in NOD2 signaling
Source: EMBO J. 2018 Jul 19;37(17):e99372. doi: 10.15252/embj.201899372 (PMC6120666; doi:10.15252/embj.201899372)
Supplement: Supplementary file 1 — Appendix [file EMBJ-37-e99372-s001.pdf]

Small-molecule inhibitors reveal an indispensable scaffolding role of RIPK2 in NOD2 signaling

APPENDIX

Matous Hrdinka[1, ¶, #], Lisa Schlicher[1, ¶], Bing Dai[2], Daniel M. Pinkas[3], Joshua C. Bufton[3, §], Sarah Picaud[3], Jennifer A. Ward[3, 4], Catherine Rogers[3, 4], Chalada Suebsuwong[5, †], Sameer Nikhar[6], Gregory D. Cuny[6], Kilian V. M. Huber[3, 4], Panagis Filippakopoulos[3], Alex N. Bullock[3], Alexei Degterev[2,*], Mads Gyrd-Hansen[1,*]

[1] Ludwig Institute for Cancer Research, Nuffield Department of Clinical Medicine, University of Oxford, Old Road Campus Research Building, Oxford OX3 7DQ, UK

[2] Department of Developmental, Molecular & Chemical Biology, Tufts University School of Medicine, 136 Harrison Avenue, Boston, MA 02111, USA

[3] Structural Genomics Consortium, Nuffield Department of Clinical Medicine, University of Oxford, Old Road Campus, Roosevelt Drive, Oxford OX3 7DQ, UK

[4] Target Discovery Institute, Nuffield Department of Clinical Medicine, University of Oxford, NDM Research Building, Roosevelt Drive, Oxford OX3 7FZ, UK

[5] Department of Chemistry, Science and Research Building 2, University of Houston, Houston, TX 77204, USA

[6] Department of Pharmacological and Pharmaceutical Sciences, Science and Research Building 2, University of Houston, Houston, TX 77204, USA

[#] Present address: University Hospital Ostrava, Department of Haematooncology, 17. listopadu 1790/5, 708 52 Ostrava-Poruba, Czech Republic

[§] Present address: Department of Biochemistry, University of Bristol, Biomedical Sciences Building, University Walk, Bristol BS8 1TH, UK

[†] Present address: Department of Pharmacological Sciences, Icahn School of Medicine at Mount Sinai, New York, NY 10029, USA

[¶] These authors contributed equally

* Corresponding authors: Mads Gyrd-Hansen, mads.gyrd-hansen@ludwig.ox.ac.uk, Alexei Degterev, alexei.degterev@tufts.edu

Running title: Mechanism of RIPK2 inhibitors

TABLE OF CONTENTS:

Appendix Figure S1, related to Figure 2

Appendix Figure Legends

Appendix Supplementary Methods

Appendix Supplementary References

Appendix Figure S1

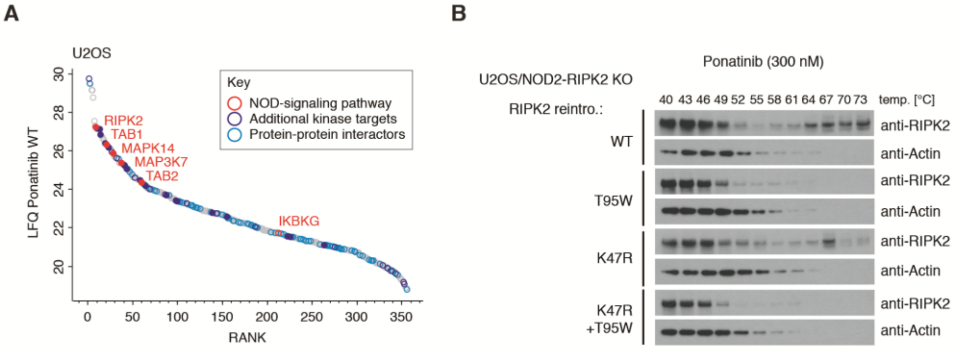

Figure S1. Cellular interactions of ponatinib determined by affinity purification and CESTA RIPK2 binding assay.

(A) Proteins enriched by c-ponatinib from U2OS cell lysate, ranked by label-free quantification (LFQ) intensity. Proteins with a CRAPome ratio < 0.2 (http://www.crapome.org/) have been further annotated to indicate NOD signaling-related targets (red), additional kinase targets (blue), and those predicted by STRING network analysis (http://string-db.org) to be enriched via protein-protein interactions (light blue). Previously reported targets of ponatinib (Canning et al., 2015; Fauster et al., 2015) are represented by a filled symbol. (This summarizes the data tabulated in supplementary data Table S1.) (B) The cellular thermal shift assay (CETSA) performed in U2OS/NOD2 RIPK2 KO cells expressing RIPK2 variants and treated with ponatinib (300 nM, 2.5 h). The amount of RIPK2 and Actin in soluble fractions were analyzed by immunoblotting.

Appendix Supplementary Methods

Plasmids and cloning

Human RIPK2 cDNA (pcDNA3-RIPK2-3xHA) was a gift from T. Tenev/P. Meier and has been described previously (Damgaard et al., 2012). Mutations of RIPK2 (K47R, D146N, T95W, R36L, R41L) were introduced by PCR-based site-directed mutagenesis of pcDNA3-RIPK2-3xHA or RIPK2 cDNA subcloned into retroviral vector pBABE-Puro (without the C-terminal 3xHA tag). Plasmids for generation of RIPK2 knockout (KO) cell lines by CRISPR/Cas9 were obtained from Santa Cruz Biotechnology, Dallas, TX (sc-400731). The XIAP BIR2 construct for production of recombinant protein for GST pull-down and probing the peptide array membranes was made by PCR cloning of XIAP BIR2 domain (aa 124-242) into pGTVL2 vector in frame with N-terminal HIS and GST tags and the D214S mutation was generated as described above. For nanoBRET experiments RIPK2 was cloned into pFC32K-Nluc (Promega, Madison, WI) and T95W mutation was introduced as described above. The NF-κB luciferase reporter plasmids pBIIX-Luc and TK-renilla-Luc and catalytically inactive XIAP (pcDNA3-3xHA-XIAP-F495A) have been described previously (Damgaard et al., 2012; Gyrd-Hansen et al., 2008).

Cell lines and cell culture conditions

NOD2-expressing U2OS-Flp-In T-REx (U2OS/NOD2) cells (Damgaard et al., 2012; Hrdinka et al., 2016) were maintained in DMEM GlutaMax (Gibco ThermoFisher Scientific, Waltham, MA) supplemented with 10 % (v/v) FBS (Sigma-Aldrich, St. Louis, MO) and 1 % (v/v) Penicillin-Streptomycin (Gibco) and cultured and stimulated in the absence of doxycycline. HEK293FT and Phoenix-A cells were cultured in DMEM GlutaMax (Gibco) supplemented with 10 % (v/v) FBS (Sigma-Aldrich) and 1 % (v/v) Penicillin-Streptomycin (Gibco). Human acute monocytic leukemia cell line THP-1 (CLS Cell Lines Service GmbH, Germany) were maintained in RPMI1640 GlutaMax medium (Gibco) supplemented with 10 % (v/v) FBS (Sigma-Aldrich), 1 % (v/v) Penicillin-Streptomycin (Gibco), 1 mM sodium pyruvate (Gibco), and 50 µM 2-mercaptoethanol (Lonza, Wakersville, CA) at density 0.6 - $1x10^6$ cells per ml. hNOD2-HEKBlue and THP1Blue cells (InvivoGen, San Diego, CA) were maintained in DMEM or RPMI1640 media with 10 % heat-inactivated FBS (Sigma-Aldrich), 1% antibiotic-antimycotic mix (ThermoFisher Scientific) and 100 mg/ml Normocin (InvivoGen)

supplemented with 30 µg/ml Blasticidin and 100 µg/ml Zeocin or 10 µg/ml Blasticidin, respectively. All antibiotics were from InvivoGen. RAW264.7 cells (ATCC, Manassas, VA) were maintained in DMEM media supplemented with 10 % heat-inactivated FBS and 1 % antibiotic-antimycotic mix.

Generation of knockout and reconstituted cell lines

To generate RIPK2 KO cells, U2OS/NOD2 cells were transfected with the CRISPR/Cas9 KO plasmids (containing gRNA, Cas9, and EGFP reporter; Santa Cruz Biotechnology) using Fugene 6 (Promega). After 36 h, top 10 % GFP-positive cells were sorted by flow cytometry and cloned by limiting dilution to obtain single cell clones. Individual clones were validated by western blotting with RIPK2 antibodies. Retroviral particles for reconstitution of RIPK2 expression in RIPK2 KO cells were generated in retroviral packaging cell line Phoenix-A. Briefly, the pBABE-Puro plasmids with RIPK2 variants were transfected into Phoenix-A cells using Fugene HD (Promega) and 72 h later virus-containing supernatants were harvested and directly used for transduction of RIPK2 KO cells in the presence of 10 µg/ml polybrene (Sigma-Aldrich) overnight. After 48 h, cells were selected with 1 µg/ml puromycin (InvivoGen) for one week.

Antibodies and immunoprecipitation reagents

The following antibodies and reagents were used according to the manufacturers' instructions. Santa Cruz Biotechnology: anti-RIPK2 mouse monoclonal (A-10, sc-166765), goat polyclonal (sc-8610) and rabbit polyclonal (H-300, sc-22763). Cell Signaling Technology (Danvers, MA): mouse monoclonal anti-Ubiquitin (clone P4D1, #3936), rabbit polyclonal anti-IκBα (#9242), rabbit anti-phospho-p65 (#3033), rabbit anti-phospho-IκBα (#2859). Merck Millipore (Temecula, CA): Mouse monoclonal anti-β-Actin (MAB1501), HRP-conjugated His-tag Antibody (His-tag® Antibody HRP conjugate kit Novagen® Merck, #71840-3). Roche (Basel, Switzerland): rat anti-HA (11867431001). Sigma-Aldrich: mouse anti-GST (G1160). HRP-conjugated secondary antibodies were from Bio-Rad, Hercules, CA (anti-rabbit) Dako Agilent Technologies, Santa Clara, CA (anti-mouse) and Santa Cruz Biotechnology (sc-2020, anti-goat). For immunoprecipitation, anti-HA-agarose conjugate (Clone HA-7, A2095, Sigma-Aldrich) was used. For intracellular staining of CXCL8 for flow cytometry the APC-conjugated mouse anti-human IL-8 Antibody (clone E8N1, BioLegend, San Diego, CA) was used.

Kinase inhibitors

The inhibitors used in this study were obtained from the following sources: ponatinib (#11494, Cayman Chemical Company, Ann Arbor, MI), GSK583 (#HY-100339, MedChem Express, South Brunswick, NJ), WEHI-345 (MedKoo Biosciences, Morrisville, NC), Compound A (CpA, a kind gift from TetraLogic Pharmaceuticals, Malvern, PA). Generation of the CSLP compound series is described in Suebsuwong *et al.* 2018 (manuscript in preparation).

hNOD2-HEKBlue and THP1Blue assays

For HEKBlue assay, cells were seeded into clear 96 well plates at $7.5x10^3$ cells/well and allowed to attach for 48 h. On the day of the experiment, media was changed to 100 µl of HEKBlue detection media (InvivoGen). Inhibitors were diluted and added in 0.5 µl DMSO 15 min prior to the addition of 1 ng/ml L18-MDP (InvivoGen). After 8-9 h, absorbance at 620 nm was measured using Victor3V plate reader (Perkin Elmer, Waltham, MA). Values of media-only wells were subtracted and %inhibition for each compound concentration relative to the DMSO/L18-MDP-treated controls were calculated. Inhibition values $\pm$ StDev were fitted by non-linear regression using Prism software (GraphPad Software, La Jolla, CA) to calculate IC_{50} values. For THP1Blue assay, $1x10^5$ cells were seeded in 200 µl of RPMI media in 96 well plates and treated with inhibitors in 0.5 µl DMSO 15 min prior to the addition of 10 µg/ml MDP, 10 ng/ml Pam3CSK4, 10 ng/ml *E. coli* LPS or $1x10^7$ cells/ml heat-killed *L. monocytogenes* (InvivoGen) for 24 h. After induction, 20 µl of media was mixed with 180 µl of QUANTI-Blue reagent (InvivoGen) and incubated at 37 °C for 3-4 h, followed by absorbance measurement at 620 nm. IC_{50} values were calculated as described for HEKBlue assay.

TNF ELISA Assay

RAW264.7 cells were seeded into 12 well plates at a density of $1.5x10^5$ cells/well in 1 ml of media. Inhibitors were diluted and added in 1 µl DMSO 15 min prior to the addition of 10 µg/ml MDP or 10 ng/ml *E. coli* LPS for 24 h. For TNF measurements, 100 µl of undiluted media (MDP) or 5-fold diluted media was analyzed using Duo-Set anti-mouse TNF ELISA kit (R&D Systems, Minneapolis, MN). Values of media-only wells were subtracted and %inhibition for each compound concentration relative to the DMSO/MDP (or LPS)-treated controls were calculated. Inhibition values $\pm$ StDev were fitted by non-linear regression using Prism software (GraphPad) to calculate IC_{50} values.

Inhibition of MDP-elicited TNF release *in vivo*

All mouse experiments were approved by the Tufts University Committee on the Use and Care of Animals and performed according to accepted veterinary standards. Female 6-8-week-old C57Bl6/J mice (Jackson labs) were used for the experiments. Experiments were performed as previously described (Nachbur et al., 2015). Tested compounds were formulated in 6% Captisol formulation, pH 4.0 (100 µl/mouse, 10 mg/kg, Ligand Pharmaceuticals, San Diego, CA). MDP (Bachem, King of Prussia, PA) was dissolved in saline (100 µl/mouse, 100 µg/mouse). Mice were administered with the drugs i.p. 30 min prior to the i.p. injections of MDP. After 4 h, blood was collected by cardiac puncture. 100 µl of serum (diluted 2-fold) per animal was analyzed using anti-mouse TNF ELISA (ThermoFisher Scientific).

Expression and purification of RIPK2 for X-ray crystallography

DNA encoding the kinase domain of human *RIPK2* (Uniprot: O43353, residues 3-317) was cloned into the transfer vector pFB-LIC-Bse, which encodes for an N-terminal hexahistidine tag and a Tobacco Etch Virus Protease A (TEV) cleavage site. Bacmid DNA was prepared in *E. coli* strain DH10Bac and used to generate baculoviruses in Sf9 insect cells. For expression, viruses were used to infect Sf9 cells grown in Insect-Xpress media (Lonza) to a density of $2x10^6$ cells/mL. Cells were harvested after 48 hours by centrifugation and resuspended in binding buffer (50 mM HEPES pH 7.4, 500 mM NaCl, 5% glycerol, 5 mM Imidazole) supplemented with protease inhibitor cocktail set III (Calbiochem, Burlington, MA) at 1:1000 dilution. Cells were lysed by sonication, 0.125% Polyethyleneimine added, clarified by centrifugation, and the recombinant protein purified by nickel-affinity chromatography eluting with increasing concentrations of imidazole in binding buffer. Fractions were pooled for further purification using a Superdex 200 26/60 gel filtration column pre-equilibrated in 50 mM HEPES pH 7.5, 300 mM NaCl, 5% glycerol, and 1 mM tris(2-carboxyethyl)phosphine (TCEP). Mass spectrometry revealed the phosphorylation state to be a mixture of between 2 to 5 phosphorylations.

Crystallization and structure determination

RIPK2 was concentrated to 10 mg/mL and 2 mM CSLP18 + 1% DMSO final concentration was added, incubated for 10 minutes, and then filtered to 0.22 um. Crystals were grown using the vapour-diffusion technique in 150 nL sitting drops containing 50 nL protein and 100 nL of a reservoir solution containing 0.2 M potassium formate and 20% (w/v) PEG3350 at 20°C. Crystals were cryo-protected

by addition of 25% ethylene glycol before being vitrified in liquid nitrogen. Diffraction data were collected at 100K on Diamond Light Source beamline I04 using 0.9795 Å light. Data were indexed and integrated using XDS (Kabsch, 2010; Leslie and Powell, 2007) and scaled using AIMLESS (Evans and Murshudov, 2013). Phases were identified using molecular replacement in PHASER (McCoy et al., 2007) and the PDB ID: 4C8B as a search model. Structures were built using PHENIX.AUTOBUILD and then refined and modified using PHENIX.REFINE (Adams et al., 2010) and COOT (Emsley et al., 2010). The refined structure was validated with MolProbity (Chen et al., 2010) and the atomic coordinate files deposited in the Protein Data Bank with Autodep (Yang et al., 2004). There were no Ramachandran outliers in the final model and 96.03% within the favored Ramachandran regions. Structure figures were prepared with PyMOL (Schrödinger, Cambridge, MA).

Purification of recombinant XIAP and cIAP1 BIR2

The recombinant WT and mutated (D214S) BIR2 domain of human XIAP or BIR2 domain of human cIAP1 was expressed in *E. coli* (BL21) cells as a fusion protein with N-terminal 6xHis (XIAP BIR2 only) and GST tags. The protein expression was induced with 500 µM IPTG for 16 h at 16 °C. Cells were lysed using BugBuster protein extraction reagent (#70921, Merck Millipore) according to manufacturer's instructions. Expressed proteins were purified from lysates in batch with Glutathione Sepharose Fast Flow (GE Healthcare Life Sciences, Little Chalfont, UK) for 4 h, washed with PBS and directly used for GST pull-down experiments or stored at 4 °C. Alternatively, cells expressing recombinant XIAP BIR2 were lysed in His6-binding buffer (50 mM HEPES pH 7.5, 500 mM NaCl, 5% Glycerol, 10 mM Imidazole) using a high-pressure homogenizer EMULSIFLEX-C5 (Avestin Europe GmbH, Germany). After centrifugation at 45000xg for 30 min at 4 °C the extract was loaded on a FPLC protein purification system ÄKTA PrimePlus (GE Healthcare Life Sciences) via a 5 ml HisTrap FF column, washed, eluted with elution buffer (50 mM HEPES pH 7.5, 500 mM NaCl, 5% Glycerol, 250 mM Imidazole), and run on a gel filtration column HiLoad 26/600 Superdex 75 pg (GE Healthcare Life Sciences) in sample buffer (50 mM HEPES pH 7.5, 300 mM NaCl, 5% Glycerol, 0.5 mM TCEP). Collected fractions corresponding to monomeric form of the recombinant protein were frozen in liquid nitrogen, stored at -80 °C and used for probing of peptide array membranes.

Immunoprecipitation, pull-downs, and purification of endogenous ubiquitin conjugates

For coimmunoprecipitation of HA-XIAP with RIPK2, transfected and treated cells were lysed in TBS buffer containing 0.5% NP-40, cOmplete and PhosSTOP inhibitors (Roche) and the lysate was cleared by centrifugation. The lysates were incubated with anti-HA-agarose overnight, washed 3x with lysis buffer and analyzed by immunoblotting. For GST pull-down experiments, U2OS/NOD2 were lysed in TBS lysis buffer containing 0.5% NP-40, cOmplete and PhosSTOP inhibitors (Roche). Cleared lysates were pretreated with kinase inhibitors or DMSO and incubated with GST-XIAP-BIR2 bound to Glutathione Sepharose at 4 °C overnight. Bound material was washed 3x with lysis buffer or PBS, eluted with 15 mM Glutathione in PBS and analyzed by immunoblotting. In pull-down experiments with recombinant (dephosphorylated) RIPK2 kinase domain (Canning et al., 2015), inhibitors or DMSO control in PBS were added together with RIPK2 kinase domain to GST-XIAP-BIR2 bound to Glutathione Sepharose. The bound material was eluted and analyzed as described above. Ubiquitin conjugates were purified from treated cells using GST-1xUBA^{ubq} ubiquitin affinity reagent and analyzed by immunoblotting as described (Fiil et al., 2013).

SPOT Peptide Assays

Cellulose-bound peptide arrays were prepared employing standard Fmoc solid phase peptide synthesis using a MultiPep-RSi-Spotter (INTAVIS, Köln, Germany) according to the SPOT synthesis method provided by the manufacturer, as previously described (Picaud and Filippakopoulos, 2015). Peptides were synthesized on amino-functionalized cellulose membranes (Whatman™ Chromatography paper Grade 1CHR, #3001-878, GE Healthcare Life Sciences) and the presence of SPOTed peptides was confirmed by ultraviolet light (UV, λ = 280 nM). The assay was performed using recombinant wild-type and mutated (D214S) 6xHIS-GST-tagged BIR2 domain of XIAP protein. Proteins bound to peptides were detected using anti-His antibody HPR conjugated (diluted 1:15000) and the Pierce® ECL Western blotting Substrate (#32106, Thermo Fisher Scientific). Chemiluminescence was detected using Super RX films (FUJIFILM, Tokyo, Japan). Peptide locations on the arrays and their sequences are given in Table S2-S4. Strong SPOTs were considered as “hits” and were further profiled employing SPOT arrays and single amino acid alanine or leucine scanning (i.e. in a given 15-mer, each position was sequentially mutated to an alanine or a leucine), and the resulting 15 peptides as well as the native sequences (control) were profiled against recombinant

GST-BIR2-XIAP as indicated. Intensity quantification of SPOTs was performed using ImageJ software (Schneider et al., 2012).

Dual luciferase reporter assay

HEK293FT cells were treated with inhibitors or DMSO control and transiently transfected with RIPK2 constructs together with the NF-κB luciferase reporter construct pBIIX-luc and a thymidine kinase-renilla luciferase construct TK-renilla-Luc for 24 h using Fugene HD transfection reagent (Promega) and reporter activity was measured on CLARIOstar microplate reader (BMG Labtech, Ortenberg, Germany) using Dual Luciferase Assay System (Promega) according to manufacturer's instructions and as previously described (Damgaard et al., 2012).

Intracellular flow cytometry of CXCL8

The cells were treated with inhibitors and stimulated with L18-MDP (InvivoGen) as indicated in figure legends. The intracellular staining of CXCL8 was performed as previously described (Hrdinka et al., 2016). The results were analyzed by FACS Canto Flow Cytometer (BD Biosciences, Franklin Lakes, NJ) and data processed using FlowJo software (FlowJo, LLC, Ashland, OR).

Viability assay

Cells were plated in 96 well plates and treated with CSLP compounds or puromycin (positive control for cell death) or vehicle for 24h. Viability was determined using the CellTiter-Glo® luminescent cell viability assay (Promega) according to manufacturer's recommendations and as previously described (Najjar et al., 2015).

RIPK2 nanoBRET assay

HEK293 cells were transiently transfected with the NanoLuc-RIPK2 WT and T95W mutant constructs together with Transfection Carrier DNA (Promega). After 20 h, transfected cells were plated at 2×10^5 cells/ml into a white 384-well assay plates (Corning, Corning, NY), treated with serial dilutions of SGC-590001, and incubated with 1 μM ponatinib or DMSO control for 3 h. After addition of Nano-Glo Substrate (Promega) and Extracellular NanoLuc Inhibitor (Promega), BRET ratios (450 nm and 610

nm) were determined using a PHERAstar FSX plate reader (BMG Labtech). The preparation of SGC-590001 is described previously (Vasta et al., 2018).

Analysis of RIPK2 cellular target occupancy and target off-rates in HEKBlue cells using nanoBRET assay

NanoLuc RIPK2 transfection. On day 1, HEKBlue cells were seeded into T-75 flask in 12 ml of media at a density of $2x10^5$ cells/ml. On day 2, 13.5 µg Transfection Carrier DNA (Promega), 1.5 µg NanoLuc-RIPK2 Vector (Promega) and 45 µL PEI (polyethylenimine) (Sigma-Aldrich) were mixed in 1.2 ml of phenol red-free Opti-MEM media (Thermo Fisher Scientific). After incubation for 30 min, DNA mixture was added to the T-75 flask containing the cells. On day 3, cells were stimulated with 1 ng/ml L18-MDP for 1 h, washed, trypsinized and resuspended in Opti-MEM media and frozen at -80 °C.

NanoBRET inhibition assay. HEKBlue cell density was adjusted to $2x10^5$ cells/ml. 100x NanoBRET In-cell Kinase Tracer (Promega) was diluted to 20x in phenol red-free Opti-MEM supplemented with 12.5 mM HEPES (Thermo Fisher Scientific) and 31.25% PEG-400 (Sigma-Aldrich). 10x inhibitor stocks in Opti-MEM were prepared by diluting DMSO stocks. For the assay, 11.9 µl cells/well were seeded into a while low volume 384 well plate (Corning) and mixed with 0.7 µl 20X tracer and 1.4 µl 10x inhibitors, followed by incubation for 2 h at 37 °C. 3x substrate mix was prepared by adding NanoBRET Nano-Glo substate (Promega) (1:166 dilution) and extracellular NanoLuc Inhibitor (Promega, 1:500 dilution) into Opti-MEM media. 7 µl of 3x substrate mix was added into each well with the cells. Plate was mixed on a rotary shaker for 15 sec at 500 rpm. Emission was determined using Victor3V plate reader at 460 nm for donor (NanoLuc) and 610 nm for acceptor (tracer). NanoBRET ratios were calculated as [($Acceptor_{sample}$ / $Donor_{sample}$) – ($Acceptor_{no\ tracer\ control}$/$Donor_{no\ tracer\ control}$)] x 1000 and used for non-linear regression to calculate IC_{50} values.

Residence time measurements using RIPK2 NanoBRET assay. HEKBlue cells transfected with NanoLuc-RIPK2 were adjusted to $2x10^5$ cells/ml and seeded into 12 well plates at 1 ml cells/well. 1 µl of each compound was added to the test wells to achieve $5xIC_{50}$ concentration determined in previous nanoBRET inhibition assay and incubated for 2h at 37 °C. After incubation, cells were washed carefully with Opti-MEM, re-adjusted to $2x10^5$ cells/ml if needed and were added to a 384 well plate at 22.5 µl/well. As positive controls for maximal inhibition, samples were left with inhibitor without

washes. 25 µl 2x substrate mix and 2.5 µl 20x tracer were added into each well. 2x substrate mix was prepared by diluting NanoBRET Nano-Glo substate (Promega, 1:250 dilution) and extracellular NanoLuc Inhibitor (Promega, 1:750 dilution) in Opti-MEM media. Plate was mixed for 10 sec at 500 rpm in a rotary shaker and the emission signals were measured 60 times with 2 min intervals. The degree of inhibition at each time point was calculated based on samples with DMSO (no inhibition) and un-washed samples with inhibitor (maximal inhibition). Percent inhibition changes over time were used for non-linear regression to calculate $t_{1/2}$ values.

Drug affinity chromatography

U2OS cells were grown until approximately 80% confluent before being pelleted and washed with PBS. Cell pellets were subsequently lysed in buffer A (50 mM Tris pH 7.5, 0.8% v/v NP-40, 5% v/v glycerol, 1.5 mM $MgCl_2$, 100 mM NaCl, 25 mM NaF, 1 mM Na_3VO_4, 1 mM PMSF, 1mM DTT, 10 µg/mL TLCK, 1 µg/ml Leupeptin, 1 µg/ml Aprotinin, 1 µg/ml soy bean trypsin) and lysates prepared and cleared by ultracentrifugation, as previously described (Fauster et al., 2015). Protein concentration was determined and lysates stored at -80 °C. A functionalized ponatinib analogue (c-ponatinib) was coupled to NHS-activated Sepharose 4 fast flow beads (GE Healthcare Life Sciences) as described previously (Fauster et al., 2015). Briefly, 100 µl of bead slurry (50% in isopropanol) were used for each pulldown experiment. Beads were washed with DMSO (3 × 500 µl), and resuspended in DMSO (50 µl), to which the amine (0.025 µmol) and triethylamine (0.75 µl) were added. The beads were incubated at room temperature for 16 h, and depletion of free amine from the supernatant was determined by LC-MS analysis. Ethanolamine (2.5 µl) was then added to block any unreacted NHS sites, and the beads incubated for a further 16 h. Derivatized beads were then washed with DMSO (3 × 500 µl), buffer A (3 × 1 ml), and incubated with cell lysates (2 mg of protein per pulldown, at 6 mg/ml) that had been pretreated with either compound (20 µM) or DMSO control for 30 min at 4 °C. Beads and treated lysates were incubated for 2 h at 4 °C before washing with buffer A (5 ml), Buffer B (50 mM HEPES pH 7.5, 100 mM NaCl, 500 µM EDTA, 2.5 ml), and eluted with formic acid (100 mM, 250 µl). Samples were neutralized with triethylammoniumbicarbonate (TEAB, 1M, 62.5 µl) and stored at -20 °C until preparation for proteomic analysis.

MS sample preparation

Samples were reduced with DTT (10 mM final concentration) for 30 min at room temperature, alkylated with iodoacetamide (55 mM final concentration) for 30 min at room temperature, diluted to 300 µl with TEAB, and incubated with trypsin (6 µl, 0.2 mg/ml) overnight at 37 °C. The digests were desalted using SEPAC lite columns (Waters), eluted with 69 % v/v MeCN, 0.1 % v/v FA in H2O (1 ml) and dried in vacuo. Dried peptides were stored at -20 °C before resuspension in 2 % v/v MeCN, 1 % v/v FA in H_2O (20 µl) for LC-MS/MS analysis.

LC-MS/MS data acquisition

Mass spectrometry data was acquired at the Discovery Proteomics Facility (University of Oxford). Digested samples were analyzed by nano-UPLC–MS/MS using a Dionex Ultimate 3000 nano UPLC with EASY spray column (75 µm × 500 mm, 2 µm particle size, Thermo Fisher Scientific) with a 60 min gradient of 0.1 % (v/v) formic acid in 5 % (v/v) DMSO to 0.1 % (v/v) formic acid with 35 % (v/v) acetonitrile in 5 % (v/v) DMSO at a flow rate of approximately 250 nl min^{-1} (600 bar per 40 °C column temperature). Mass spectrometry data was acquired either with an Orbitrap Q Exactive (Survey scans acquired at a resolution of 70,000 @ 200m/z and the 15 most abundant precursors were selected for HCD fragmentation), or an Orbitrap Q Exactive High Field (HF) instrument (survey scans were acquired at a resolution of 60,000 at 400 m/z and the 20 most abundant precursors were selected for CID fragmentation).

MS data analysis

Raw data was processed using MaxQuant version 1.5.0.253 and the reference complete human proteome FASTA file (Uniprot). Label Free Quantification (LFQ) and Match Between Runs were selected; replicates were collated into parameter groups to ensure matching between replicates only. Cysteine carbamidomethylation was selected as a fixed modification, and methionine oxidation as a variable modification. Default settings for identification and quantification were used. Specifically, a minimum peptide length of 7, a maximum of 2 missed cleavage sites, and a maximum of 3 labelled amino acids per peptide were employed. Peptides and proteins were identified utilizing a 0.01 false discovery rate, with “Unique and razor peptides” mode selected for both identification and quantification of proteins (razor peptides are uniquely assigned to protein groups and not to individual proteins). At least 2 razor + unique peptides were required for valid quantification. Processed data

was further analyzed using Perseus version 1.5.0.9 and Microsoft Excel 2010. Peptides categorized by MaxQuant as ‘potential contaminants’, ‘only identified by site’ or ‘reverse’ were filtered, and the LFQ intensities transformed by Log2. Experimental replicates were grouped, and 2 valid LFQ values were required in at least one experimental group. Missing values were imputed using default settings, and the data distribution visually inspected to ensure that a normal distribution was maintained. Statistically significant competition was determined through the application of P2 tests, using a permutation-based FDR of 0.05 and an S0 of 2, and visualized in volcano plots. Significantly competed targets were further analyzed in STRING (http://string-db.org) and protein interaction networks generated.

Docking Experiments

Ligand docking of CSLP37 and CSLP43 were performed using AutoDock molecular docking software (version 4.2.6) (http://autodock.scripps.edu/) with standard protocol. Co-crystal structure of CSLP18 bound to RIPK2 was used for docking experiment where CSLP18 was extracted from the RIPK2 by using Discovery Studio 2016 Client software (http://accelrys.com/). CSLP37 and CSLP43 ligands were created and subjected to energy minimization using MM2 force field. Grid maps were selected based on CSLP18-RIPK2 co-crystal structure and docking experiments were performed. The final pose was selected based on the lowest binding energy and were analyzed using PyMOL, a molecular visualization software (https://pymol.org/2/).

Data deposition

The accession number for the coordinates and structure factors for the RIPK2 kinase domain bound by CSLP18 is PDB: 6FU5.

Statistical analysis

Statistical analysis and calculation of IC_{50} values was performed using Prism 6 (GraphPad Software). Unpaired Student t-test and two-way ANOVA were used to determine statistical significance as appropriate.

Appendix Supplementary References

Adams, P.D., Afonine, P.V., Bunkoczi, G., Chen, V.B., Davis, I.W., Echols, N., Headd, J.J., Hung, L.W., Kapral, G.J., Grosse-Kunstleve, R.W.*, et al.* (2010). PHENIX: a comprehensive Python-based system for macromolecular structure solution. Acta Crystallogr D Biol Crystallogr *66*, 213-221.

Canning, P., Ruan, Q., Schwerd, T., Hrdinka, M., Maki, J.L., Saleh, D., Suebsuwong, C., Ray, S., Brennan, P.E., Cuny, G.D.*, et al.* (2015). Inflammatory Signaling by NOD-RIPK2 Is Inhibited by Clinically Relevant Type II Kinase Inhibitors. Chem Biol *22*, 1174-1184.

Chen, V.B., Arendall, W.B., 3rd, Headd, J.J., Keedy, D.A., Immormino, R.M., Kapral, G.J., Murray, L.W., Richardson, J.S., and Richardson, D.C. (2010). MolProbity: all-atom structure validation for macromolecular crystallography. Acta Crystallogr D Biol Crystallogr *66*, 12-21.

Damgaard, R.B., Nachbur, U., Yabal, M., Wong, W.W., Fiil, B.K., Kastirr, M., Rieser, E., Rickard, J.A., Bankovacki, A., Peschel, C.*, et al.* (2012). The ubiquitin ligase XIAP recruits LUBAC for NOD2 signaling in inflammation and innate immunity. Mol Cell *46*, 746-758.

Emsley, P., Lohkamp, B., Scott, W.G., and Cowtan, K. (2010). Features and development of Coot. Acta Crystallogr D Biol Crystallogr *66*, 486-501.

Evans, P.R., and Murshudov, G.N. (2013). How good are my data and what is the resolution? Acta Crystallogr D Biol Crystallogr *69*, 1204-1214.

Fauster, A., Rebsamen, M., Huber, K.V., Bigenzahn, J.W., Stukalov, A., Lardeau, C.H., Scorzoni, S., Bruckner, M., Gridling, M., Parapatics, K.*, et al.* (2015). A cellular screen identifies ponatinib and pazopanib as inhibitors of necroptosis. Cell Death Dis *6*, e1767.

Fiil, B.K., Damgaard, R.B., Wagner, S.A., Keusekotten, K., Fritsch, M., Bekker-Jensen, S., Mailand, N., Choudhary, C., Komander, D., and Gyrd-Hansen, M. (2013). OTULIN restricts Met1-linked ubiquitination to control innate immune signaling. Mol Cell *50*, 818-830.

Gyrd-Hansen, M., Darding, M., Miasari, M., Santoro, M.M., Zender, L., Xue, W., Tenev, T., da Fonseca, P.C., Zvelebil, M., Bujnicki, J.M., *et al.* (2008). IAPs contain an evolutionarily conserved ubiquitin-binding domain that regulates NF-kappaB as well as cell survival and oncogenesis. Nat Cell Biol *10*, 1309-1317.

Hrdinka, M., Fiil, B.K., Zucca, M., Leske, D., Bagola, K., Yabal, M., Elliott, P.R., Damgaard, R.B., Komander, D., Jost, P.J., *et al.* (2016). CYLD Limits Lys63- and Met1-Linked Ubiquitin at Receptor Complexes to Regulate Innate Immune Signaling. Cell Rep *14*, 2846-2858.

Kabsch, W. (2010). Xds. Acta Crystallogr D Biol Crystallogr *66*, 125-132.

Leslie, A.G.W., and Powell, H.R. (2007). Processing diffraction data with MOSFLM. Nato Sci Ser Ii-Math *245*, 41-+.

McCoy, A.J., Grosse-Kunstleve, R.W., Adams, P.D., Winn, M.D., Storoni, L.C., and Read, R.J. (2007). Phaser crystallographic software. J Appl Crystallogr *40*, 658-674.

Nachbur, U., Stafford, C.A., Bankovacki, A., Zhan, Y., Lindqvist, L.M., Fiil, B.K., Khakham, Y., Ko, H.J., Sandow, J.J., Falk, H., *et al.* (2015). A RIPK2 inhibitor delays NOD signalling events yet prevents inflammatory cytokine production. Nature communications *6*, 6442.

Najjar, M., Suebsuwong, C., Ray, S.S., Thapa, R.J., Maki, J.L., Nogusa, S., Shah, S., Saleh, D., Gough, P.J., Bertin, J., *et al.* (2015). Structure guided design of potent and selective ponatinib-based hybrid inhibitors for RIPK1. Cell Rep *10*, 1850-1860.

Picaud, S., and Filippakopoulos, P. (2015). SPOTing Acetyl-Lysine Dependent Interactions. Microarrays (Basel) *4*, 370-388.

Schneider, C.A., Rasband, W.S., and Eliceiri, K.W. (2012). NIH Image to ImageJ: 25 years of image analysis. Nature methods *9*, 671-675.

Vasta, J.D., Corona, C.R., Wilkinson, J., Zimprich, C.A., Hartnett, J.R., Ingold, M.R., Zimmerman, K., Machleidt, T., Kirkland, T.A., Huwiler, K.G., *et al.* (2018). Quantitative, Wide-Spectrum Kinase Profiling in Live Cells for Assessing the Effect of Cellular ATP on Target Engagement. Cell Chem Biol *25*, 206-214 e211.

Yang, H., Guranovic, V., Dutta, S., Feng, Z., Berman, H.M., and Westbrook, J.D. (2004). Automated and accurate deposition of structures solved by X-ray diffraction to the Protein Data Bank. Acta Crystallogr D Biol Crystallogr *60*, 1833-1839.
