# Supplementary material for: Small molecule inhibitors reveal an indispensable scaffolding role of RIPK2 in NOD2 signaling
Source: EMBO J. 2018 Jul 19;37(17):e99372. doi: 10.15252/embj.201899372 (PMC6120666; doi:10.15252/embj.201899372)
Supplement: Supplementary file 2 — Expanded View Figures PDF [file EMBJ-37-e99372-s002.pdf]

Expanded View Figures

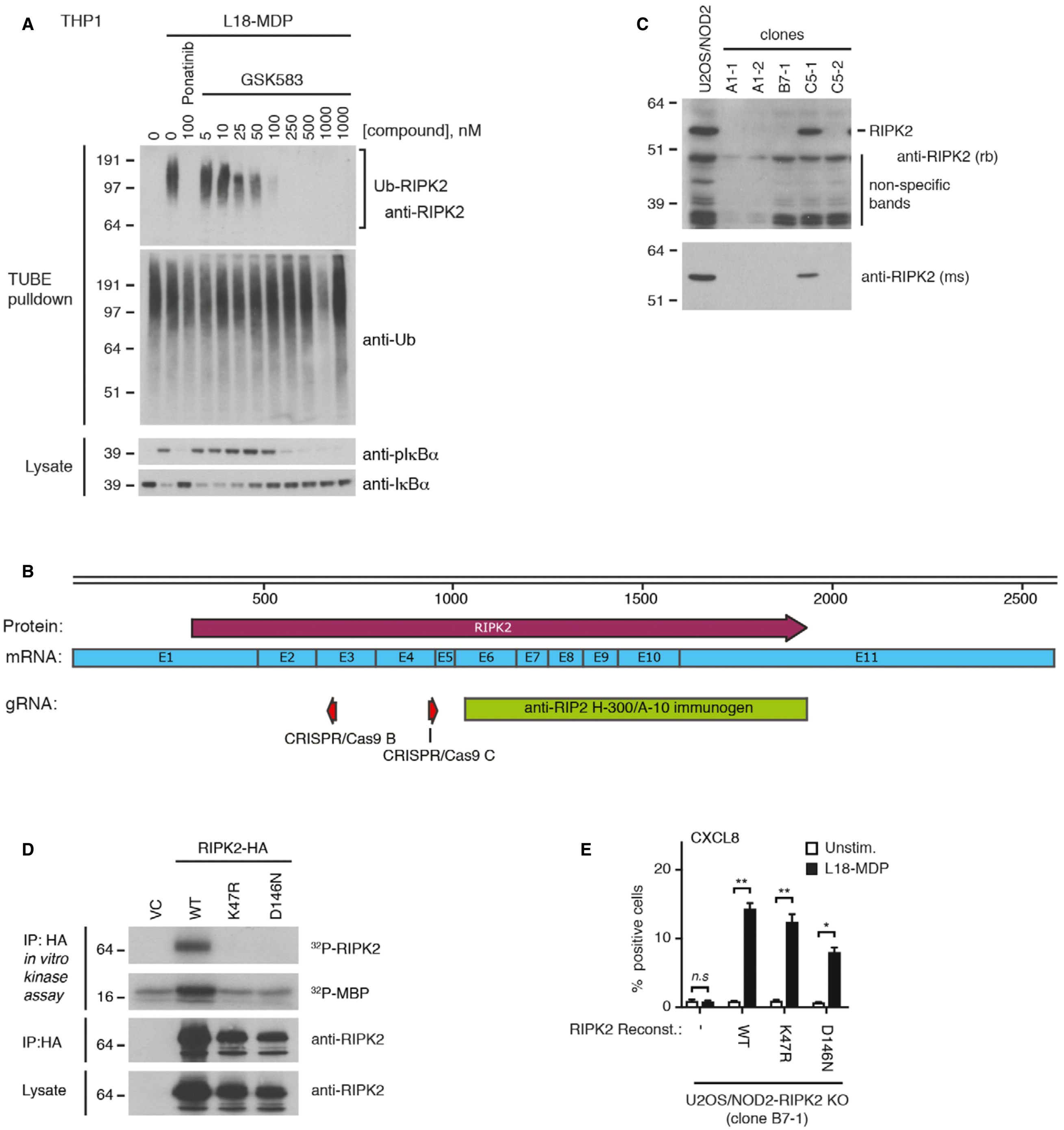

Figure EV1.

Figure EV1. RIPK2 kinase-dead mutants support NOD2 signaling.

A Purification of Ub-conjugates from THP-1 cells after treatment with L18-MDP (200 ng/ml, 1 h) and ponatinib or GSK583 as indicated. Purified material and lysates were analyzed by immunoblotting.

B Schematic representation of RIPK2 mRNA and protein with indicated positions of RIPK2 gRNA sequences (CRISPR/Cas9 B and C) and epitopes for RIPK2 antibodies used in this study. E1–E11 indicates RIPK2 exons.

C Identification RIPK2 knockout U2OS/NOD2 cell clones grown from cell cultures transfected with CRISPR/Cas9 vectors targeting RIPK2. Clones B7-1 and C5-2 were used in the study.

D Radioactive *in vitro* RIPK2 kinase assay with RIPK2 variants expressed in U2OS/NOD2 RIPK2 KO cells and purified with anti-HA. The *in vitro* phosphorylated RIPK2 and universal kinase substrate MBP were separated by SDS–PAGE and exposed to X-ray film. The inputs and precipitated proteins were analyzed by immunoblotting.

E Intracellular flow cytometry analysis of CXCL8 following L18-MDP treatment (200 ng/ml, 4 h) of U2OS/NOD2 RIPK2 KO cells (clone B7-1) reconstituted with RIPK2 variants or empty vector as indicated.

Data information: Data represent the mean ± SEM of at least three independent experiments. *$P < 0.05$, **$P < 0.01$, n.s., not significant. Two-way ANOVA was used to determine statistical significance.

Source data are available online for this figure.

Figure EV2. Structural basis and selectivity of CSLP mode of RIPK2 inhibition.

A Intracellular flow cytometry analysis of CXCL8 of U2OS/NOD2 cells treated with L18-MDP (200 ng/ml, 4 h) and CSLP inhibitors as indicated.

B Viability of U2OS/NOD2, THP1, RAW264.7, and HEKBlue cells treated with CSLP inhibitors for 24 h. Cell viability was determined using CellTiter-Glo assay (Promega). Puromycin (2 or 10 μg/ml) was used as a positive control for cell death.

C CSLP37 and CSLP43 do not inhibit recombinant RIPK1 and RIPK3 kinases. Kinase activities were determined using ADPGlo assay (Promega).

D Structure of the active conformation of RIPK2 kinase domain in complex with AMP-PNP (PDB ID: 5AR3). Figure depicts locations of the adenine and deep pockets. Alignment of two active AMP-PNP-bound conformations of RIPK2 (5AR3, green sticks; 5NG0, brown sticks) with CSLP18-bound RIPK2 (blue sticks) reveals its inactive conformation. Key residues discussed in the text are shown as sticks (Lys/Glu Glu-in bond—bottom left, catalytic Asp residues (Asp146/Asp164)—bottom right, R-spine—right). P-loop of CSLP18 structure is shown in yellow. Structured segment of activation segment (AS) observed in active conformation structures is shown in red.

E Structure of RIPK2 kinase domain (gray ribbons) in complex with CSLP18 (orange) (PDB ID 6FU5). Sticks are shown for residues in the vicinity of the cavity surrounding the key inhibitor position R1. A 2Fo-Fc electron density map contoured at 1 sigma is shown for the inhibitor and the residues lining the key region around inhibitor R^1 position.

F Molecular docking model of RIPK2 kinase domain in complex with CSLP37 (yellow) based on RIPK2/CSLP18 structure (Fig 3D). Key residues from CSLP18/RIPK2 structure and residues forming "R^1" pocket, Ala45, Lys47, Ile93, Thr95 are shown as sticks.

G Intracellular flow cytometry analysis of CXCL8 of U2OS/NOD2 RIPK2 KO cells reconstituted retrovirally with RIPK2 wt or T95W mutant treated with L18-MDP (200 ng/ml, 4 h) and CSLP inhibitors as indicated. Values represent the percent CXCL8-positive cells relative to L18-MDP treatment for each RIPK2 variant without inhibitor treatment.

Data information: Data in (A–C, G) represent the mean ± SEM of three independent experiments. **$P < 0.01$. Two-way ANOVA was used to determine statistical significance.

© 2018 The Authors

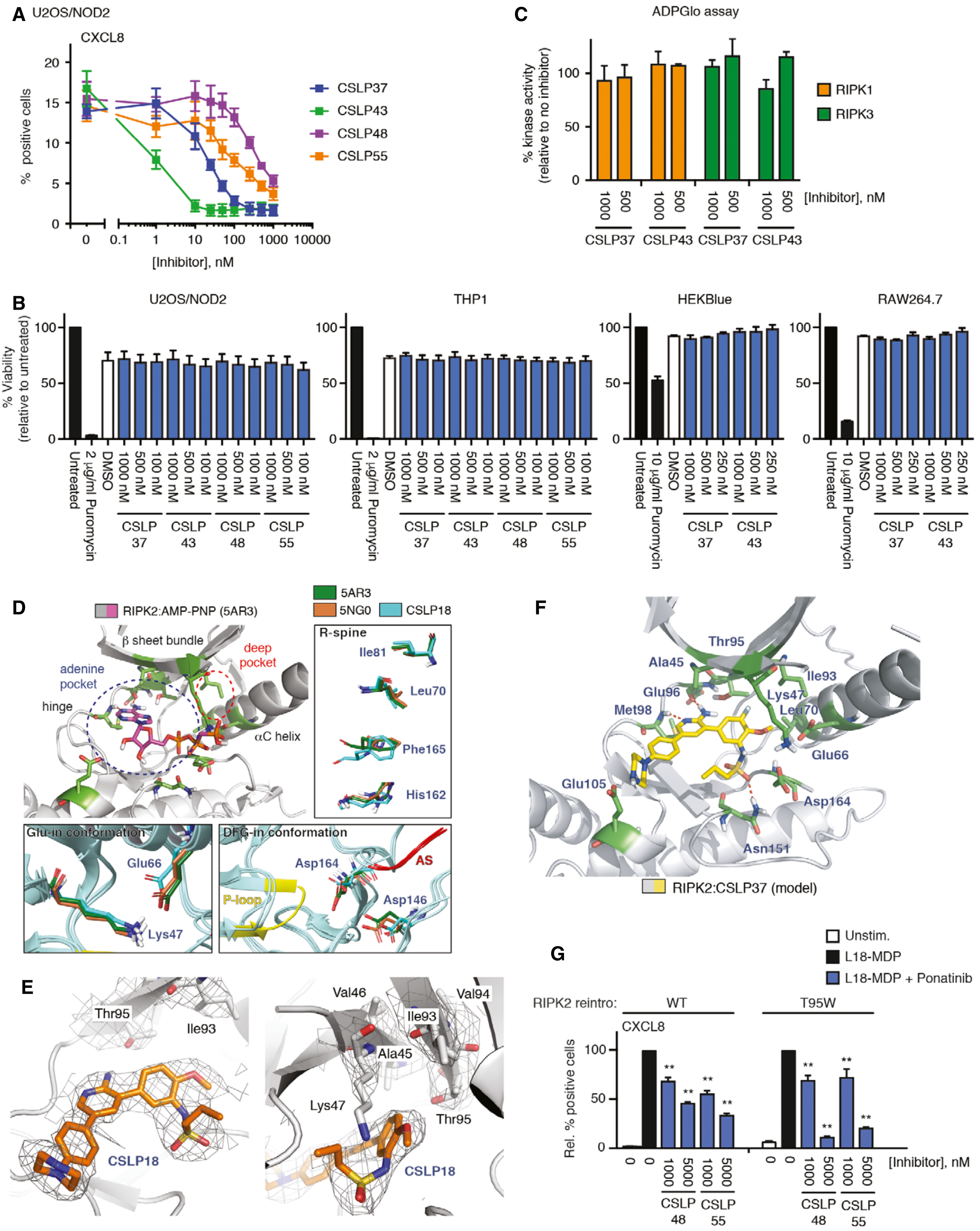
A U2OS/NOD2
CXCL8
% positive cells
[Inhibitor], nM
CSLP37
CSLP43
CSLP48
CSLP55
C ADPGlo assay
% kinase activity (relative to no inhibitor)
RIPK1
RIPK3
[Inhibitor], nM
CSLP37 CSLP43 CSLP37 CSLP43
B U2OS/NOD2
THP1
HEKBlue
RAW264.7
% Viability (relative to untreated)
Untreated
2 µg/ml Puromycin
10 µg/ml Puromycin
DMSO
CSLP 37
CSLP 43
CSLP 48
CSLP 55
D RIPK2:AMP-PNP (5AR3)
5AR3
5NG0
CSLP18
β sheet bundle
adenine pocket
deep pocket
hinge
αC helix
R-spine
Ile81
Leu70
Phe165
His162
Glu-in conformation
Glu66
Lys47
DFG-in conformation
Asp164
AS
P-loop
Asp146
E Thr95
Ile93
CSLP18
Val46
Val94
Ala45
Lys47
F Thr95
Ala45
Ile93
Glu96
Lys47
Met98
Leu70
Glu66
Glu105
Asp164
Asn151
RIPK2:CSLP37 (model)
G Unstim.
L18-MDP
L18-MDP + Ponatinib
RIPK2 reintro:
WT
T95W
CXCL8
Rel. % positive cells
[Inhibitor], nM
CSLP 48
CSLP 55

Figure EV2.

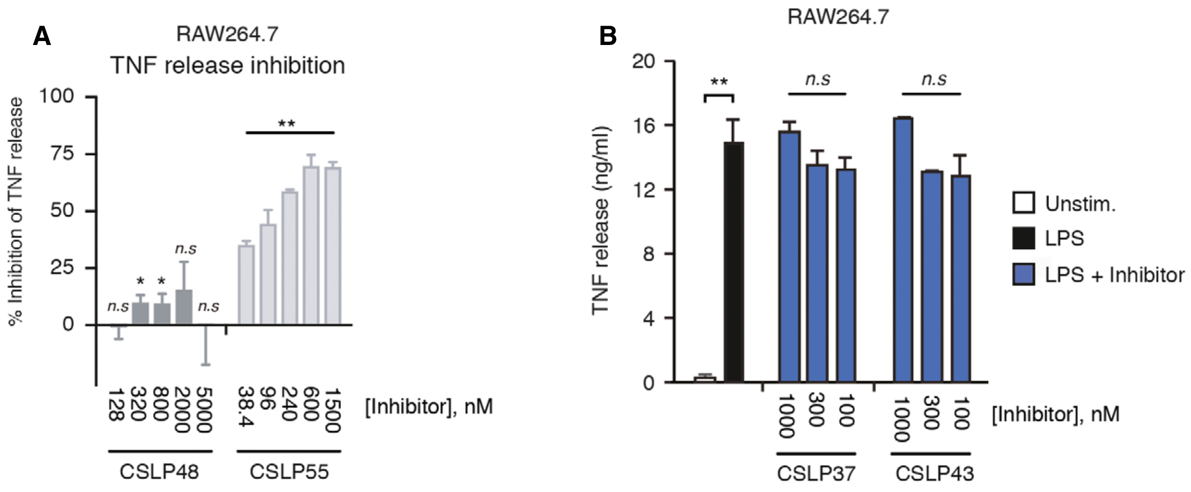

Figure EV3. Activity of select CSLP inhibitors in MDP and LPS-stimulated RAW264.7 macrophages.

A, B ELISA measurement of TNF release from RAW264.7 cells treated with MDP (10 μg/ml, 24 h) (A) or LPS (10 ng/ml) (B) and CSLP compounds as indicated. Values are expressed relative to the TNF release in cells treated with MDP without inhibitor (A) or as ng/ml TNF (B).

Data information: Data represent the mean ± SEM of 2–3 independent experiments. Statistical significance in (B) is determined in relation to L18-MDP-stimulated samples without inhibitor *$P < 0.05$, **$P < 0.01$, n.s., not significant. Two-tailed unpaired Student's *t*-test was used to determine statistical significance.

© 2018 The Authors

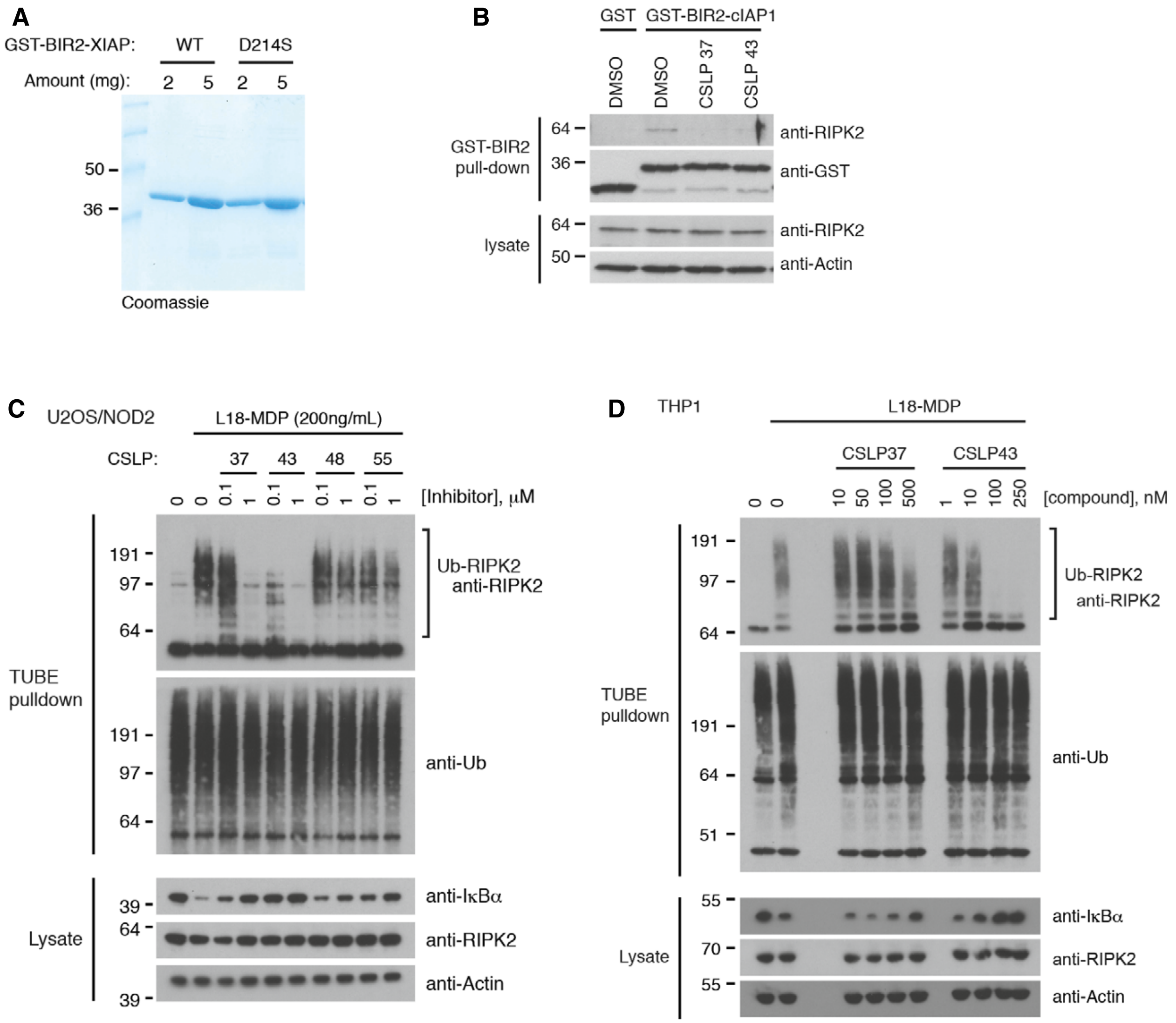

Figure EV4. Inhibition of RIPK2 ubiquitination and cIAP1 binding by CSLP inhibitors.

A Coomassie Blue staining of His Trap and gel filtration purified His6-GST-XIAP-BIR2 WT and D214S recombinant proteins at two different concentrations as indicated.

B Pulldown of RIPK2 from U2OS/NOD2 cell lysates with recombinant GST-BIR2-cIAP1 in the presence of CSLP inhibitors. Inhibitors were used at 100-fold IC_{50} of RIPK2 kinase activity: CSLP 37 (1.8 μM), 43 (2 μM). Purified material and lysates were analyzed by immunoblotting.

C, D Purification of Ub-conjugates from U2OS/NOD2 cells (C) or THP-1 cells (D) after treatment with L18-MDP (200 ng/ml, 1 h) and CSLP compounds as indicated. Purified material and lysates were analyzed by immunoblotting.

Source data are available online for this figure.

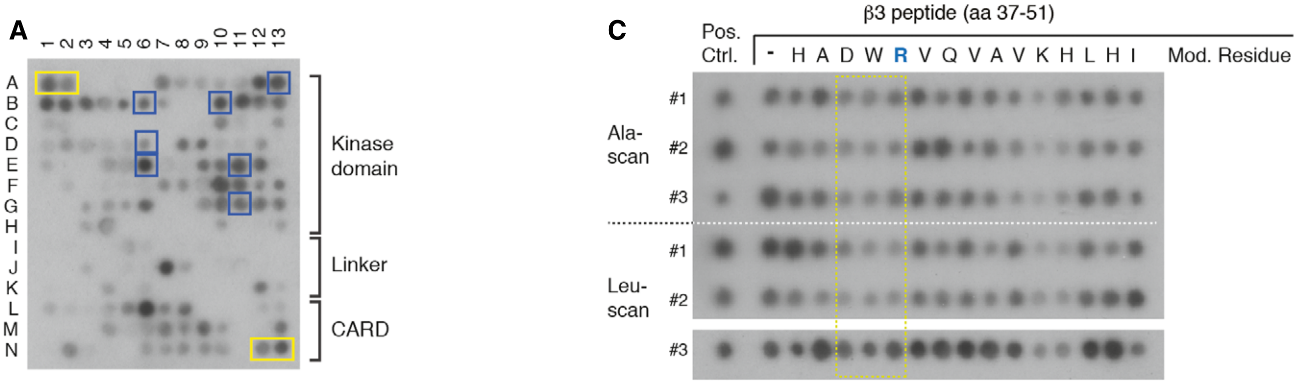

B

A13 (28-42): A-S-G-T-V-S-S-A-R-H-A-D-W-R-V	β2-loop
B6 (46-60): V-K-H-L-H-I-H-T-P-L-L-D-S-E-R	Loop-αC helix
B10 (58-72): S-E-R-K-D-V-L-R-E-A-E-I-L-H-K	αC helix
D6 (124-138): I-L-H-E-I-A-L-G-V-N-Y-L-H-N-M	αD helix (C-lobe)
E6 (163-177): A-D-F-G-L-S-K-W-R-M-M-S-L-S-Q	Activation segment
E11 (178-192): S-R-S-S-K-S-A-P-E-G-G-T-I-I-Y	Activation segment
G11 (256-270): P-Y-D-I-P-H-R-A-R-M-I-S-L-I-E	αG helix (C-lobe)

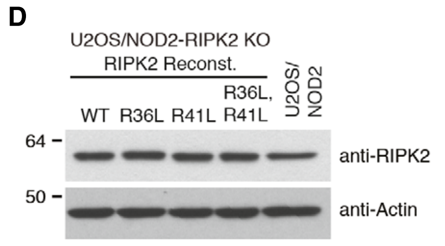

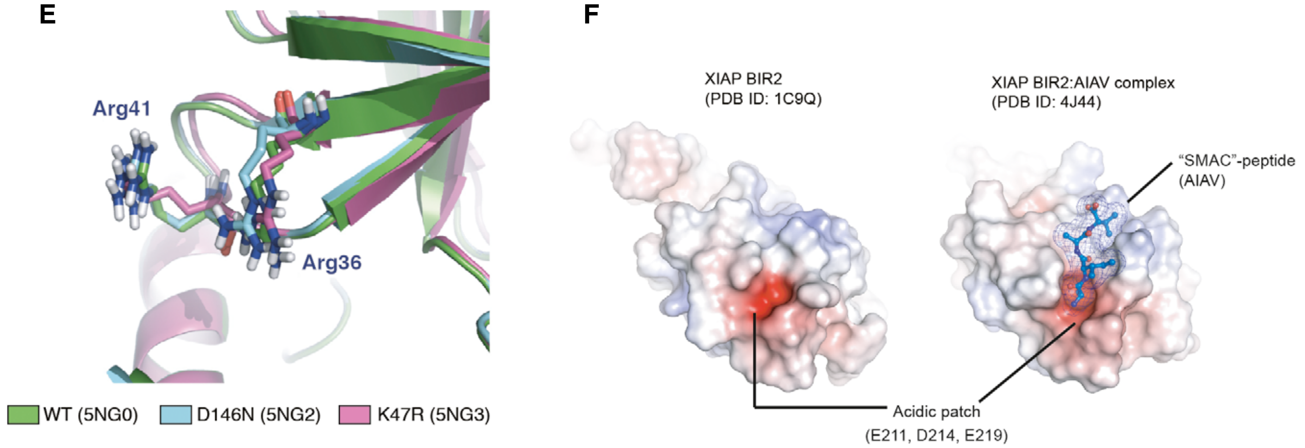

Figure EV5. Mapping BIR2 XIAP - RIPK2 binding interface.

A SPOT peptide assay to probe interaction of recombinant GST-BIR2-XIAP with partially overlapping RIPK2 peptides (15-mers) immobilized to the membrane. The bound BIR2 domain was visualized by HRP-conjugated anti-His. Seven potentially interacting peptides (blue boxes) were selected for further validation. 11xHis peptides were spotted as a positive control for anti-His-HRP staining (yellow boxes).

B Table shows the position on membrane shown in (A), amino acid sequence and RIPK2 residue ranges, and structural features of the corresponding region in RIPK2, of the peptides selected for further validation.

C SPOT peptide array of a peptide corresponding to β3 in RIPK2 (aa 37–51) in which each residue is substituted with alanine (Ala-scan) or leucine (Leu-scan) was probed for binding of GST-XIAP BIR2. Each peptide was spotted in triplicate indicated by #1–#3. Yellow box indicates the residues in peptide A13 where binding is reduced by modification.

D U2OS/NOD2 RIPK2 KO cells reconstituted with RIPK2 variants by retroviral transduction. Cell lysates were analyzed for RIPK2 levels by immunoblotting to confirm equal expression of variants.

E Alignment of the crystal structure of the wild-type RIPK2 (5NG0) and D146N (5NG2) and K47R (5NG3) mutants reveals that R36/41 XIAP binding area retains the same conformation.

F Surface charge representation of XIAP-BIR2 (left) (PDB ID: 1C9Q) or XIAP-BIR2 in complex with a SMAC-like peptide (AIAV; right) (PDB ID: 4J44). AIAV peptide is represented by sticks with space-filling mesh. The peptide binds in the IBM groove overlapping with the acidic patch bound by RIPK2.

Source data are available online for this figure.

 © 2018 The Authors
