# Supplementary material for: Small molecule inhibitors reveal an indispensable scaffolding role of RIPK2 in NOD2 signaling
Source: EMBO J. 2018 Jul 19;37(17):e99372. doi: 10.15252/embj.201899372 (PMC6120666; doi:10.15252/embj.201899372)
Supplement: Supplementary file 3 — Table EV1 [file EMBJ-37-e99372-s003.docx]

**Table EV1.** **Data collection and refinement statistics**

|  | RIPK2-CSLP18 |  |  |
| --- | --- | --- | --- |
| **Data collection** |  |  |  |
| Space group | P2_1_2_1_2_1_ |  |  |
| Cell dimensions |  |  |  |
| *a*, *b*, *c* (Å) | 75.79, 122.72, 71.41 |  |  |
| α, β, γ (°) | 90, 90, 90 |  |  |
| Resolution (Å) | 39.66 – 3.26 (3.38 – 3.26) |  |  |
| *R*_merge_ | 0.1748 (1.41) |  |  |
| *I* / σ*I* | 7.12 (1.17) |  |  |
| Completeness (%) | 99.48 (99.44) |  |  |
| Redundancy | 6.0 (6.0) |  |  |
|  |  |  |  |
| **Refinement** |  |  |  |
| Resolution (Å) | 39.66 – 3.26 (3.38 – 3.26) |  |  |
| No. reflections | 10837 (1060) |  |  |
| *R*_work_  *R*_free_ | 0.2292 (0.3705)  0.2710 (0.4053) |  |  |
| No. non-H atoms | 4634 |  |  |
| Protein | 4557 |  |  |
| Ligand/ion | 68 |  |  |
| Water | 9 |  |  |
| *B*-factors | 94.67 |  |  |
| Protein | 94.78 |  |  |
| Ligand/ion | 90.97 |  |  |
| Water | 69.86 |  |  |
| R.m.s. deviations |  |  |  |
| Bond lengths (Å) | 0.004 |  |  |
| Bond angles (°) | 0.95 |  |  |
