# Supplementary figures and images for: Small molecule inhibitors reveal an indispensable scaffolding role of RIPK2 in NOD2 signaling
Source: EMBO J. 2018 Jul 19;37(17):e99372. doi: 10.15252/embj.201899372 (PMC6120666; doi:10.15252/embj.201899372)

Figure EV1

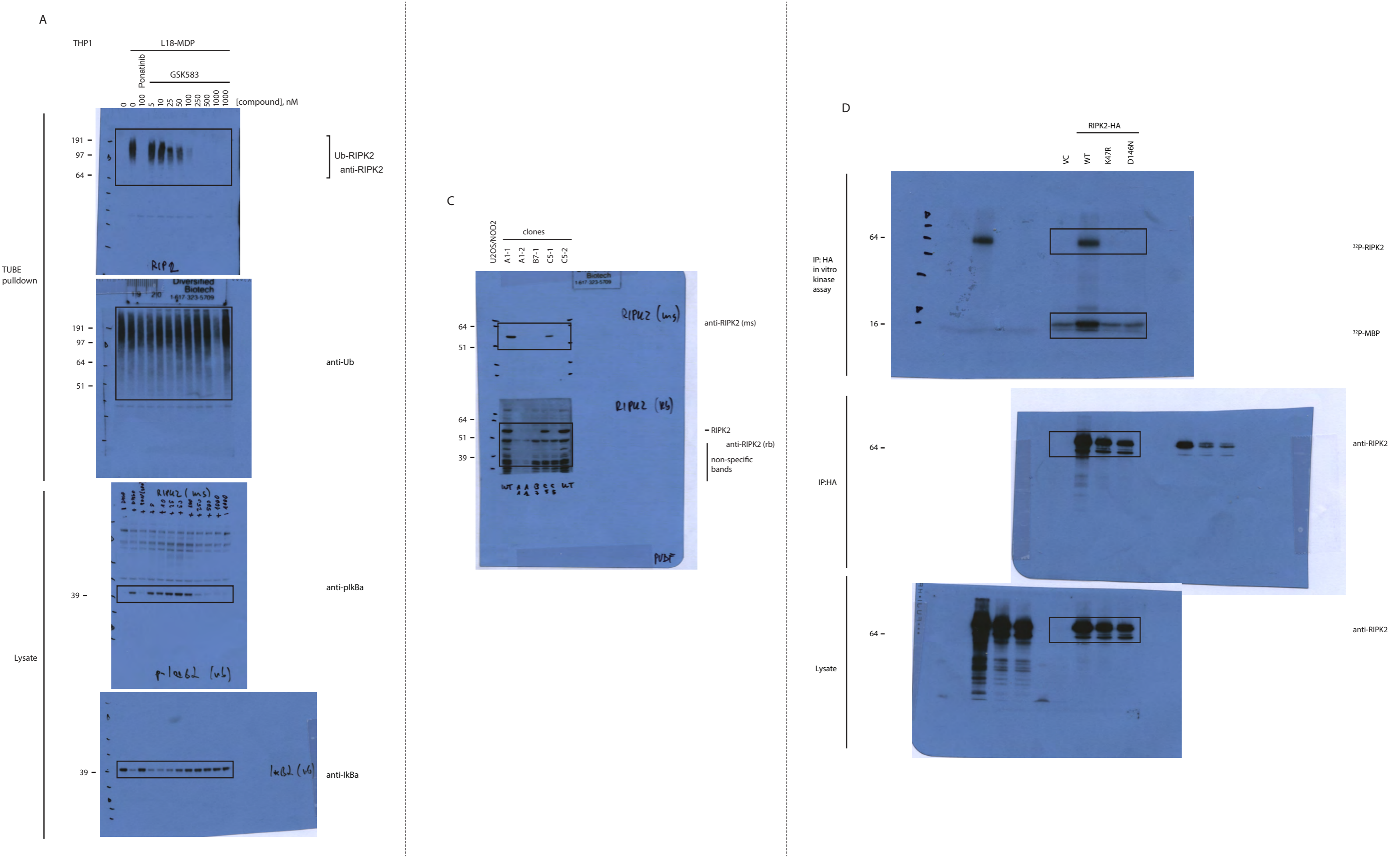

Supplement: Supplementary file 8 — Source Data for Expanded View [file EMBJ-37-e99372-s012.zip › EMBOJ-2018-99372_SourceDataForFigEV1.pdf]

Figure EV4

B

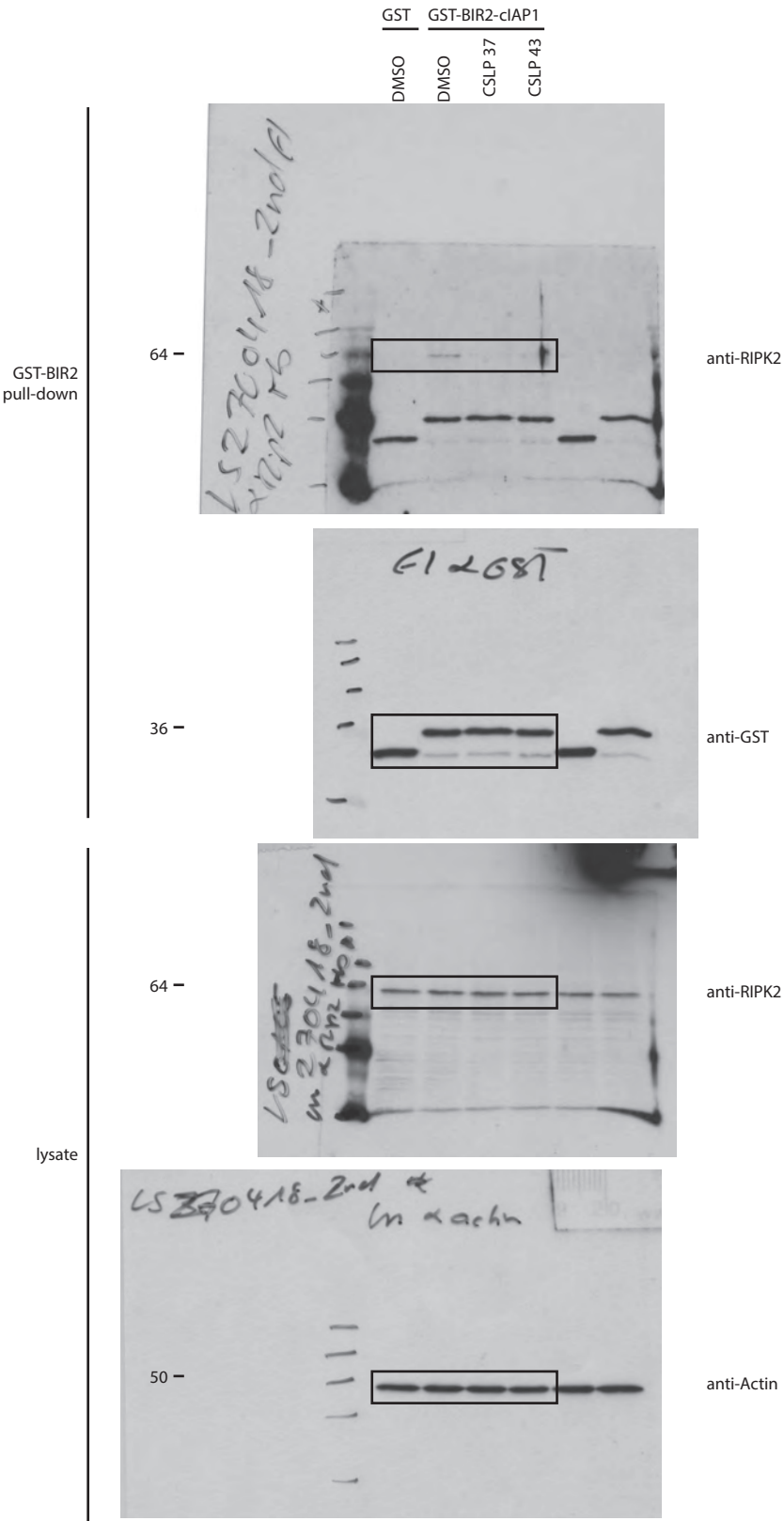

C

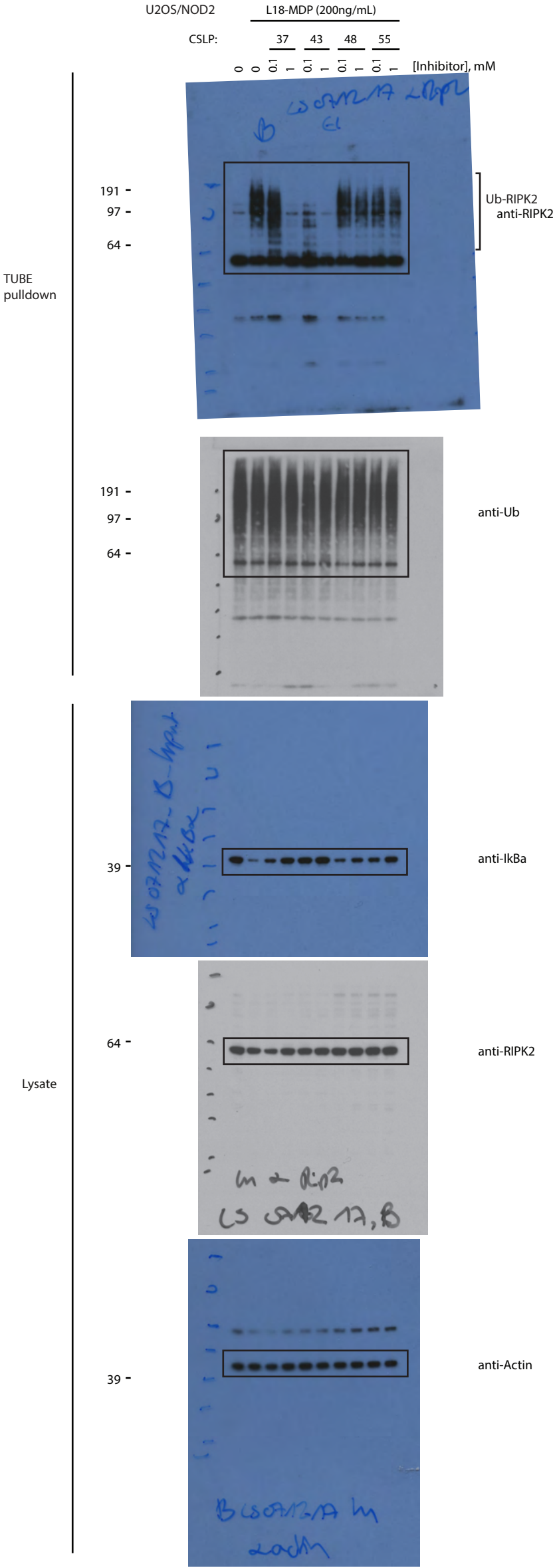

D

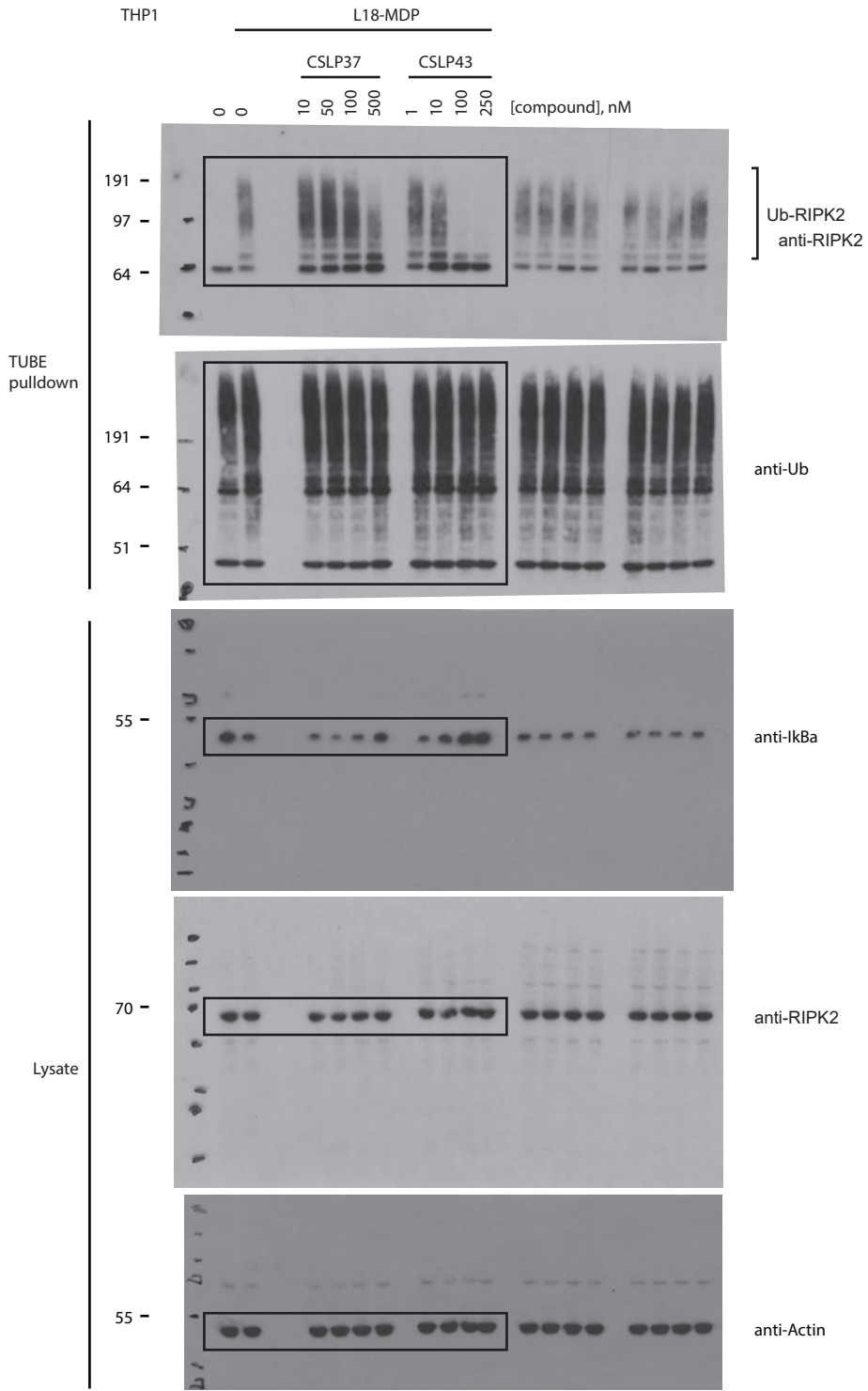

Supplement: Supplementary file 8 — Source Data for Expanded View [file EMBJ-37-e99372-s012.zip › EMBOJ-2018-99372_SourceDataForFigEV4.pdf]

Figure EV5

D

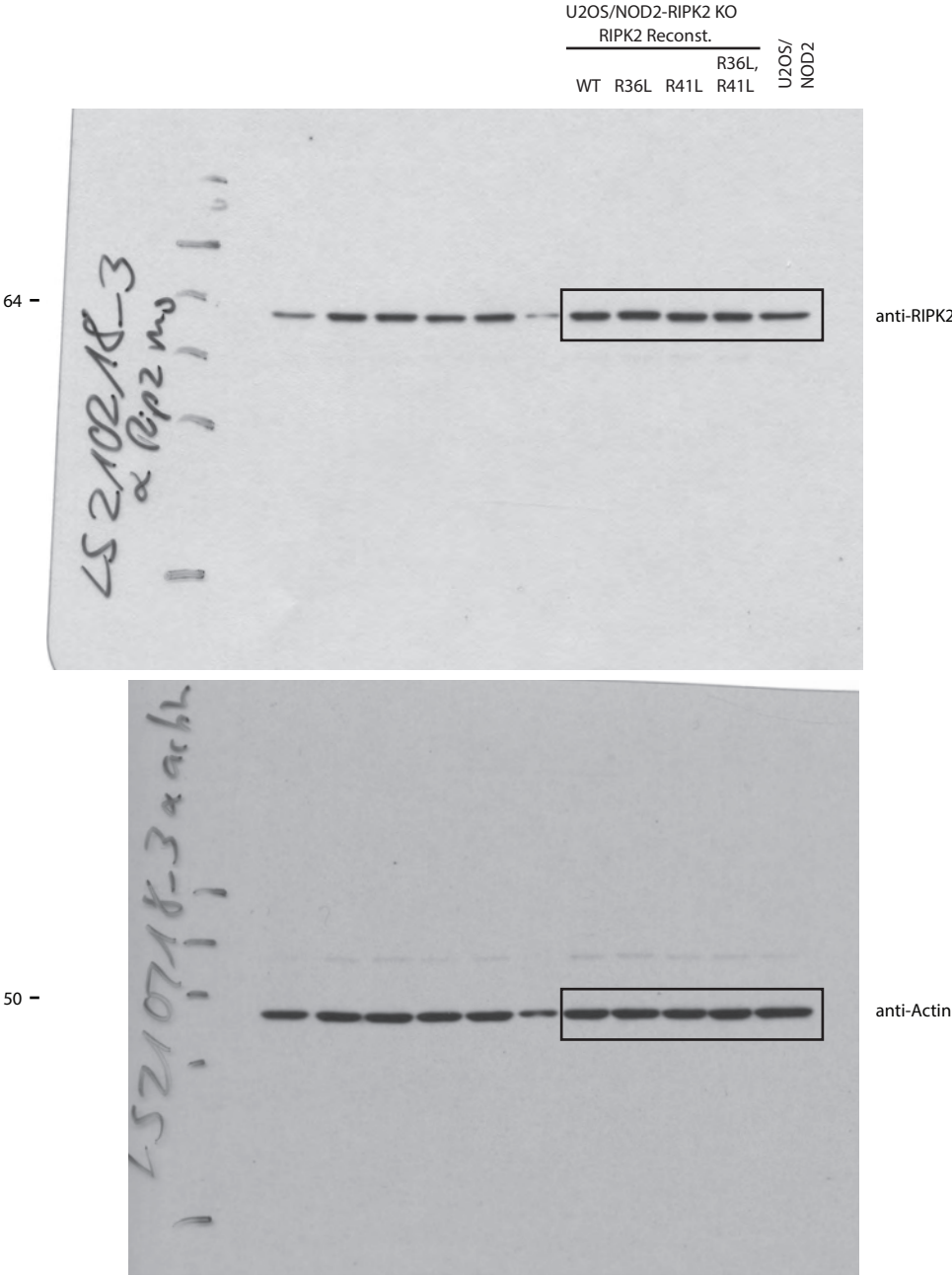

Supplement: Supplementary file 8 — Source Data for Expanded View [file EMBJ-37-e99372-s012.zip › EMBOJ-2018-99372_SourceDataForFigEV5.pdf]

Figure 1

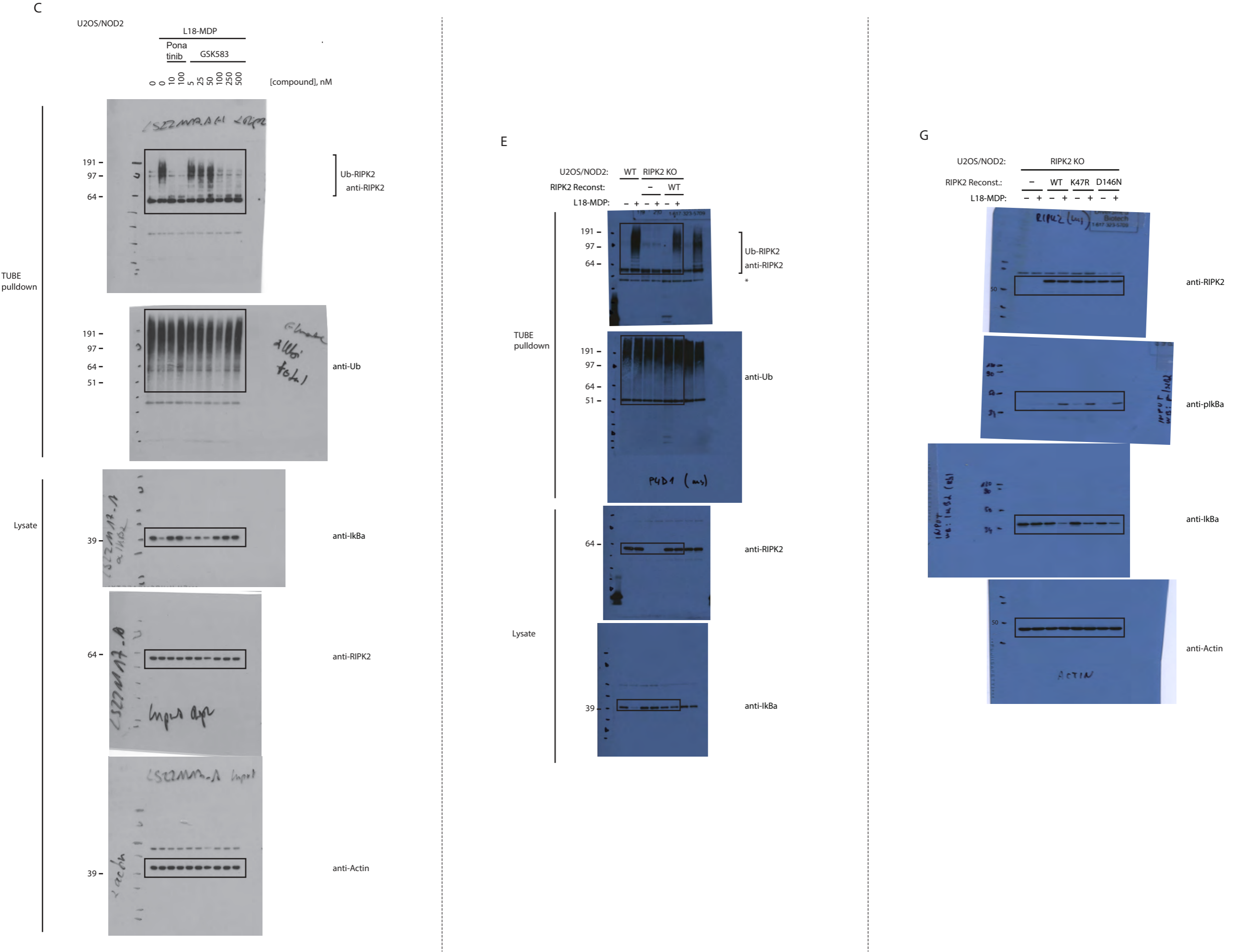

Supplement: Supplementary file 10 — Source Data for Figure 1 [file EMBJ-37-e99372-s008.pdf]

Figure 2

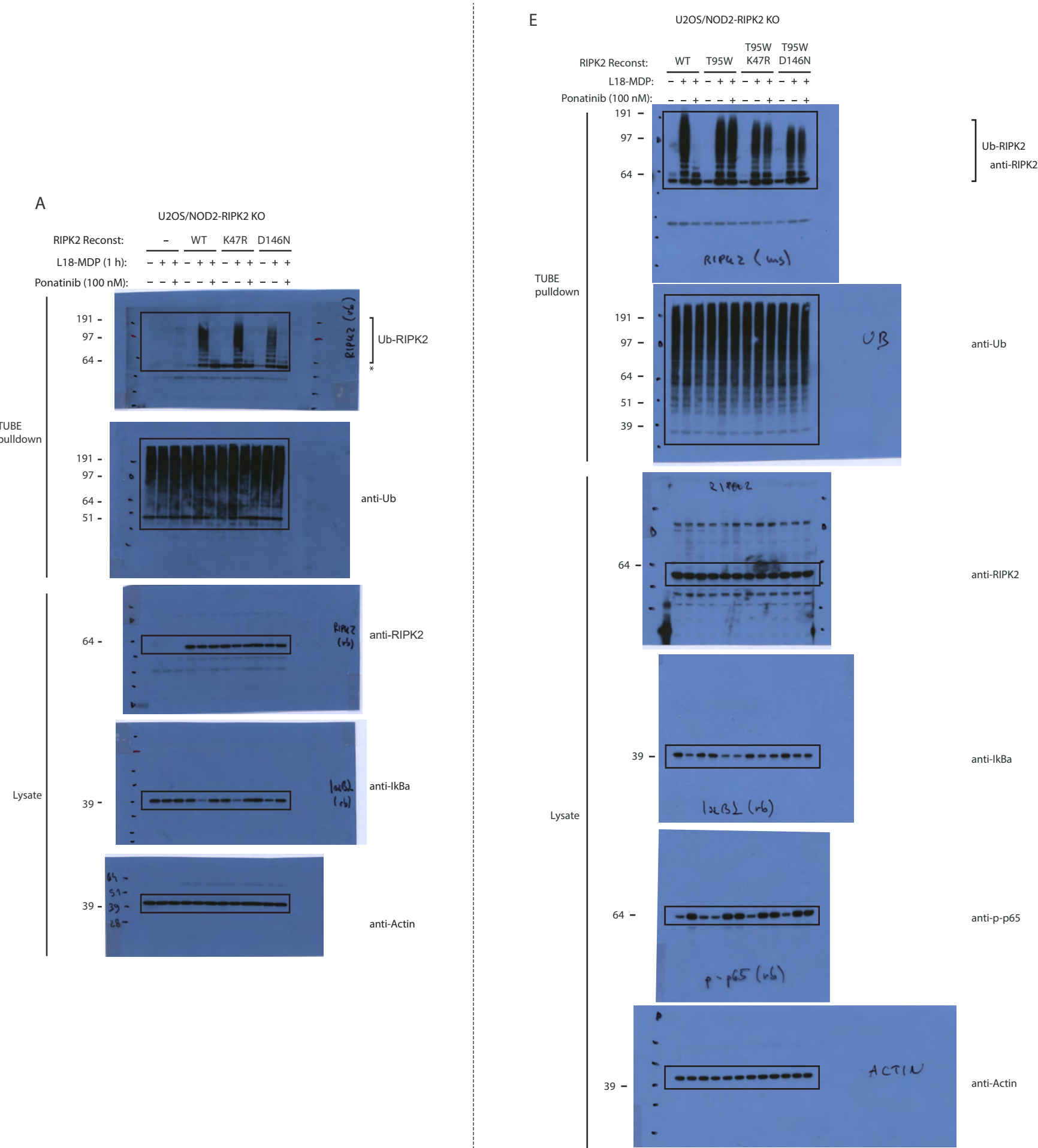

Supplement: Supplementary file 11 — Source Data for Figure 2 [file EMBJ-37-e99372-s009.pdf]

Figure 5

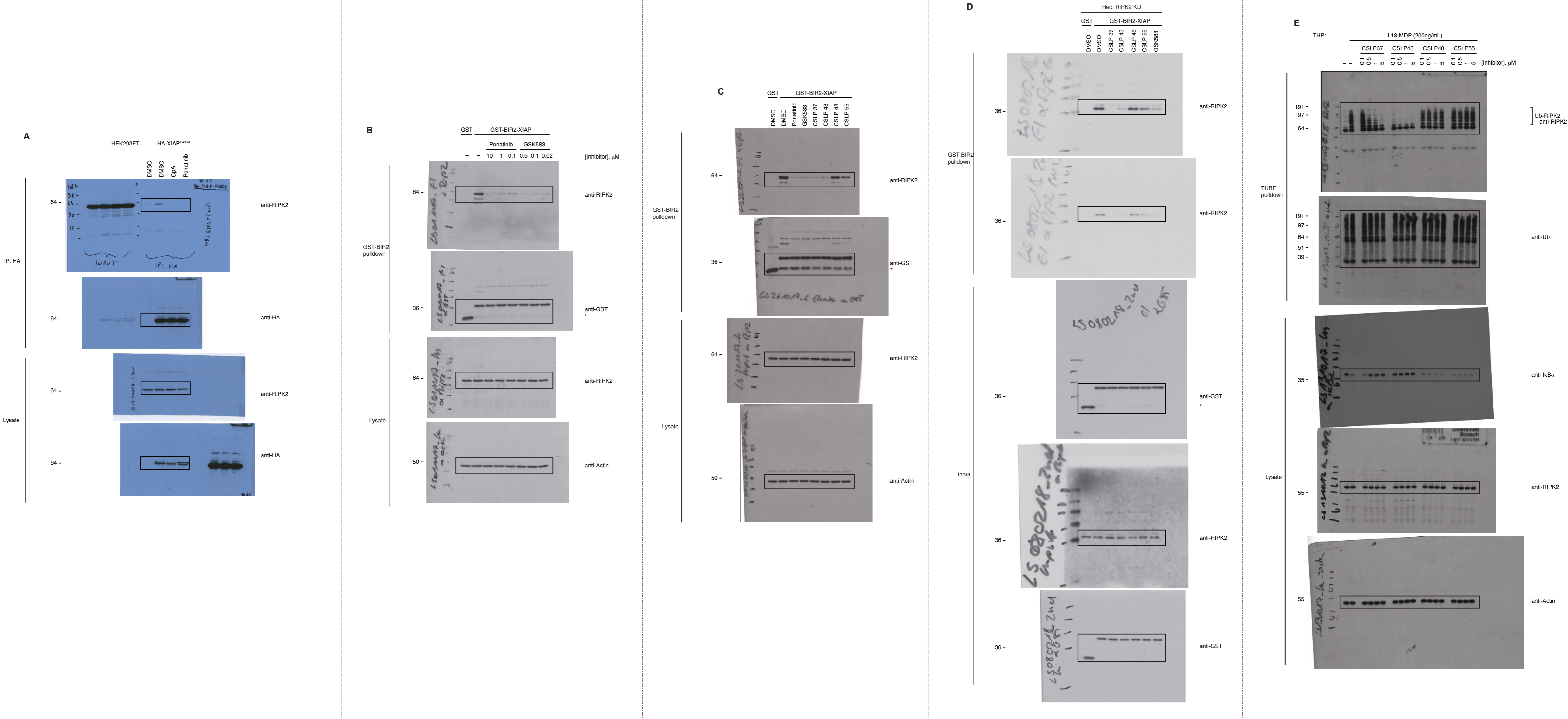

Supplement: Supplementary file 12 — Source Data for Figure 5 [file EMBJ-37-e99372-s010.pdf]

Figure 6

F

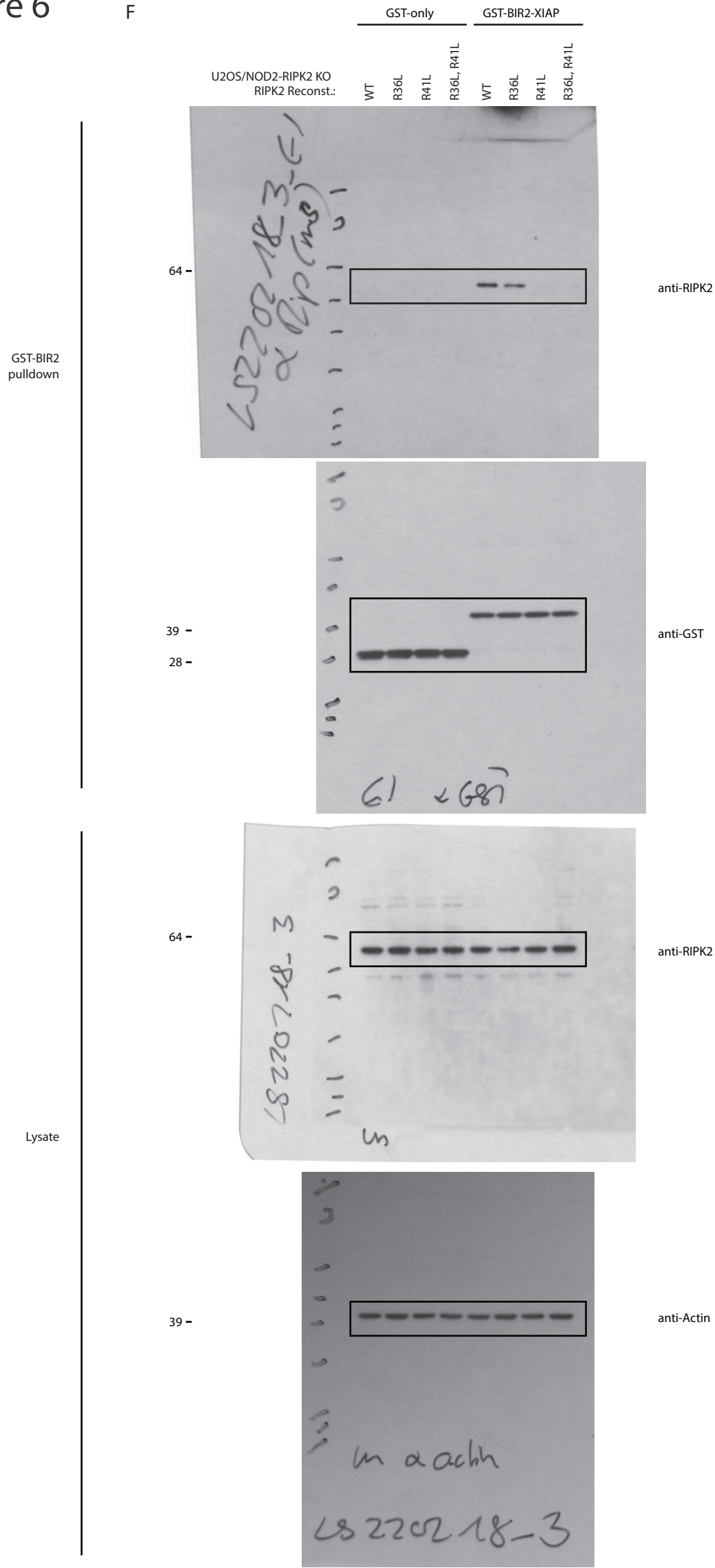

Supplement: Supplementary file 13 — Source Data for Figure 6 [file EMBJ-37-e99372-s011.pdf]
